# Microenvironment awareness pragmatic analysis for generative enzyme function reasoning

**DOI:** 10.1093/bioinformatics/btag769

**Published:** 2026-10-13

**Authors:** Jing Hao, Ke Yan, Bin Liu

**Affiliations:** School of Computer Science and Technology, Beijing Institute of Technology, Beijing 100081, China; Zhongguancun Academy, Beijing 100094, China; School of Computer Science and Technology, Beijing Institute of Technology, Beijing 100081, China; Zhongguancun Academy, Beijing 100094, China; School of Computer Science and Technology, Beijing Institute of Technology, Beijing 100081, China; Zhongguancun Academy, Beijing 100094, China

## Abstract

**Motivation:**

Accurate enzyme function annotation is critical for interpreting rapidly expanding sequence data, yet experimental methods are costly and low-throughput, while computational approaches face two key limitations. Most existing methods are discriminative models confined to known EC labels, making it difficult to generalize to emerging enzymatic activities under cold-start conditions. Moreover, they typically treat enzymes as isolated molecular entities and overlook microenvironment factors such as optimal pH and temperature, which provide important contextual clues for functional annotation.

**Results:**

This study proposes Microenvironment awareness pragmatic analysis for generative enzyme function reasoning, a generative framework that integrates microenvironment as pragmatic context into enzyme function reasoning. By fine-tuning the large language model on a newly constructed Enzyme Microenvironment Database, the model learns to infer microenvironment from sequence and then reason function within that context, generating EC labels with explicit reasoning chains. Multi-task enzyme microenvironment prediction imputes missing pH and temperature values to complete the Enzyme Microenvironment Database. On public benchmark dataset, Microenvironment Awareness Pragmatic Analysis for Generative Enzyme Function Reasoning achieves an absolute F1 improvement of 0.1068 over the state-of-the-art GraphEC model. Under cold-start conditions, the model generates reference-worthy function labels, demonstrating its practical utility for emerging enzymatic activities.

**Availability and implementation:**

The source code and data of MPA-EC can be accessed at https://bliulab.net/MPA-EC, https://zenodo.org/records/22152329 and https://github.com/bliulab/MPA-EC.

## 1 Introduction

Enzymes are biomolecules that catalyze biochemical reactions with high efficiency and specificity, playing vital roles in cellular metabolism (Kaelin Jr and Thompson 2010), energy conversion (Gao *et al.* 2006), industrial manufacturing (Clomburg *et al.* 2017), and pharmaceutical research (Li *et al.* 2025). Enzymes are numerous and possess complex catalytic functions, which researchers classify and annotate using a four-digit Enzyme Commission (EC) numbering system (Barrett 1997). Although traditional experimental EC numbers annotation approaches such as enzyme kinetics analysis (Yu, *et al.* 2023), X-ray crystallography(James 1980), and mass spectrometry (Bothner *et al.* 2000) are accurate and reliable, their high cost and low throughput make it difficult to address the challenges posed by the rapidly growing petabyte-scale sequence data. For example, in mainstream public databases such as UniProt, known protein sequences exceed 253 million entries, but less than 0.3% have undergone rigorous manual review and annotation (The UniProt Consortium 2025). Specifically, enzymes possessing novel catalytic functions beyond the scope of known enzyme families continue to be created and discovered, driven by the development of enzyme engineering (Sun *et al.* 2025) and high-throughput experimental screening (Jing *et al.* 2025). Therefore, leveraging computational approaches to accurately and efficiently annotate unknown or novel catalytic functions has become a fundamental and urgent task, which is crucial for advancing enzyme engineering and enzyme discovery.

Currently, computational methods can be categorized into rule-based and machine learning-based approaches. Rule-based methods (Pearson and Lipman 1988; Altschul *et al.* 1990, 1997; Krogh *et al.* 1994; Wang *et al.* 2010; Desai *et al.* 2011; Roy *et al.* 2012; Buchfink *et al.* 2015; Steinegger *et al.* 2019) infer enzyme functions using predefined rules, typically relying on the assumption that high sequence similarity implies similar functions. Tools including BLAST (Altschul *et al.* 1990) are commonly employed for sequence alignment. When similarity is low, the reliability of predictions diminishes. Consequently, subsequent methods integrate structural similarity (Wang *et al.* 2010, Roy *et al.* 2012) and homology information (Krogh *et al.* 1994, Desai *et al.* 2011, Steinegger *et al.* 2019), such as sequence conservation, to enhance accuracy. However, these methods remain constrained by the coverage of available comparable templates. Machine learning-based methods (Dalkiran *et al.* 2018, Li *et al.* 2018, Ryu *et al.* 2019, Sarker *et al.* 2020, Han *et al.* 2023, Sanderson *et al.* 2023, Yu *et al.* 2023, Song *et al.* 2024, van der Weg *et al.* 2025) typically extract feature representations from enzyme sequences and subsequently train corresponding prediction models. Traditional methods rely on manually designed feature extraction, then construct classifiers by integrating multiple prediction models such as multi-label *k*-nearest neighbors algorithms (Zou and Xiao 2016). With the advancement of deep learning, researchers employ protein language models (e.g. ESM (Rives *et al.* 2021) and ProtTrans (Elnaggar *et al.* 2022)) to automatically obtain high-dimensional sequence embeddings and leverage deep neural networks to learn feature representations for enzyme function prediction. For instance, CLEAN (Yu *et al.* 2023) optimizes protein language model embeddings through contrastive learning (Heinzinger *et al.* 2022), enhancing the discriminative power of sequence representations. DeepEC (Ryu *et al.* 2019), ECPICK (Han *et al.* 2023), ProteInfer (Sanderson *et al.* 2023), and DEEPre (Li *et al.* 2018) use convolutional neural networks, recurrent neural networks, or hybrid architectures to learn functional representations from sequences. GrAPFI (Sarker *et al.* 2020) performs label propagation based on domain similarity graphs. To overcome the limitations of sequence-only information, recent methods further integrate structural data. GraphEC (Song *et al.* 2024) combines ProtTrans language model embeddings with ESMFold-predicted (Lin *et al.* 2023) structures for prediction via geometric graph learning, and TopEC (van der Weg *et al.* 2025) constructs local 3D descriptors and leverages 3D graph neural networks (Kipf and Welling 2017) to directly learn functional information from enzyme structures. Although the above methods have made some progress in enzyme functional annotation, they still face two fundamental limitations. First, most current approaches are discriminative models with annotation scope limited to known functional label sets, making it difficult to provide reference-worthy annotations for catalytic functions beyond the training set distribution. Second, existing models often treat enzymes as isolated molecular entities during computational modeling, with limited consideration of the complex biological microenvironments, such as optimal pH (pH) and temperature, that serve as important contextual clues for functional realization.

On the one hand, advances in high-throughput screening and enzyme engineering are generating enzymes with potentially novel activities, some of which are not yet cataloged in existing databases. For example, according to the IUBMB proposed changes to the enzyme list (IUBMB, 2026), over 25 new EC numbers were added in the June 2026 update (International Union of Biochemistry and Molecular Biology n.d.). Such frequent updates highlight the growing need for rapid and accurate annotation. However, discriminative models operate as classifiers with a fixed output space defined by known EC labels, which limits their ability to generalize to previously unseen functions, a “cold start” problem. In contrast, generative large language models perform open-vocabulary reasoning and are not confined to a predefined label set. They can infer the sequence–function relation from learned prior knowledge and generate reference-worthy EC labels, thus offering superior generalization to emerging enzymatic activities.

On the other hand, enzyme function is determined not only by sequence and structure, but also by microenvironment factors such as pH and temperature, etc., (Koshland 1958, Zhang *et al.* 2017). For example, as shown in Fig. 1, two homologous enzymes, DNA dC→dU-editing enzyme APOBEC-3G (UniProt: Q7YR25, EC 3.5.4.38) and C→U-editing enzyme APOBEC-2 (UniProt: Q9WV35, EC 3.5.4.36), belong to the same cytidine deaminase family and share 46.9% sequence identity, yet catalyze different reactions under distinct microenvironmental conditions (Q7YR25: pH 5.5, 37°C; Q9WV35: pH 7.4, 45°C). These observations suggest that microenvironment factors can serve as useful clues for distinguishing among related catalytic activities. To incorporate such clues into EC prediction, we construct an enzyme microenvironment database integrating sequences, EC numbers, reactions, and pH/temperature values, partially sourced from BRENDA (Schomburg *et al.* 2004, Chang *et al.* 2021) and UniProt (Bairoch *et al.* 2005) databases, with missing data imputed by prediction models. Several computational tools (Ferrer *et al.* 2007; Zhang *et al.* 2009; Gado *et al.* 2020, 2025; Li *et al.* 2020; Verma and Satyanarayana 2020; Shahraki *et al.* 2022; Qiu *et al.* 2025) have been developed for microenvironment prediction, such as EpHod (Gado *et al.* 2025) or pH and Seq2Topt[50] for optimal temperature (Tem). However, these models make isolated single-parameter predictions without considering intrinsic correlations among microenvironment features. To overcome this, we propose Multi-task enzyme microenvironment prediction (MEMP), a multi-task framework that jointly predicts multiple microenvironment parameters and completes missing database entries.

**Figure 1:**
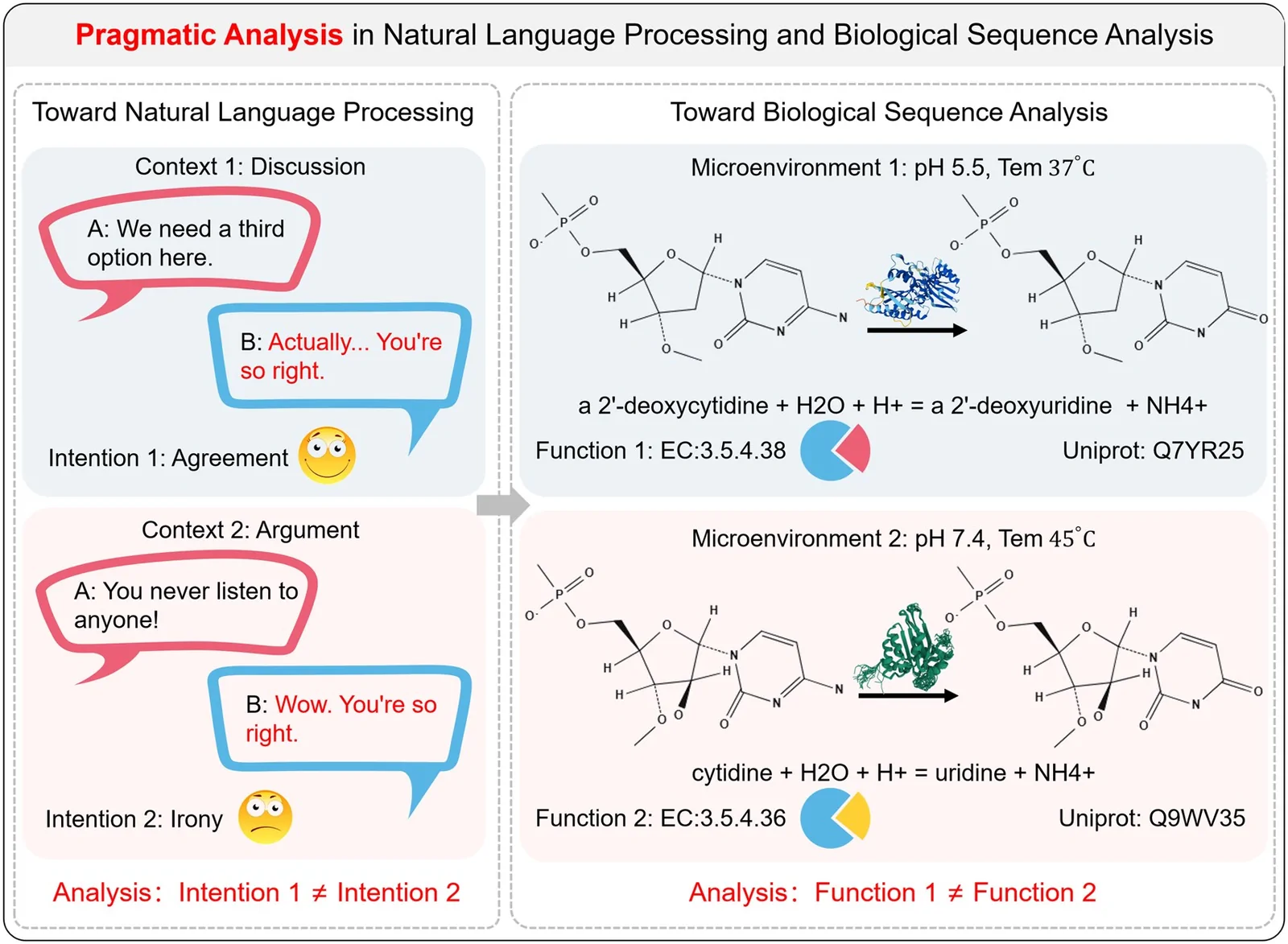
Pragmatic analysis analogy between natural language processing and biological sequence analysis. In natural language processing, the identical sentence “You’re so right” conveys distinct pragmatic meanings depending on the context, expressing agreement in a discussion setting but irony in an argument setting. In biological sequence analysis, two homologous enzymes, DNA dC→dU-editing enzyme APOBEC-3G (Q7YR25, EC 3.5.4.38) and C→U-editing enzyme APOBEC-2 (Q9WV35, EC 3.5.4.36), which belong to the same cytidine deaminase family and share 46.9% sequence identity, yet catalyze different reactions and exist under distinct microenvironment conditions (Q7YR25: pH 5.5, 37°C; Q9WV35: pH 7.4, 45°C ). This comparison suggests that microenvironment can serve as an important clue for functional annotation.

Effectively incorporating microenvironment information into generative reasoning with large language models for enzyme annotation is a key challenge. Advances in natural language processing (NLP) offer valuable insights, as an isomorphism exists between natural language and biological sequences: both are composed of fundamental units (words or amino acids) arranged in specific sequences. Critically, in both cases, meaning is shaped not only by the individual units themselves but also by their surrounding context, as illustrated in Fig. 1. The concept of pragmatic analysis (Bach 1997, Kamp 2013) in NLP, which examines how meaning is shaped by the context of usage, directly aligns with the challenge of context-aware enzyme annotation. While IIDL-PepPI (Chen *et al.* 2025) has applied pragmatic analysis to peptide–protein interactions using implicit context vectors, we propose Microenvironment Awareness Pragmatic Analysis for Generative Enzyme Function Reasoning (MPA-EC), which integrates microenvironment into an explicit “sequence—microenvironment—function” reasoning process. Our work fine-tunes LLaMA-3.1-8B via Low-Rank Adaptation (LoRA) (Hu *et al.* 2022) on the enzyme microenvironment database to inject this causal logic, enabling the model to generate reference-worthy EC labels with explicit reasoning chains.

To summarize, this study proposes the Microenvironment Awareness Pragmatic Analysis for generative enzyme function annotation. Its core scientific innovation lies in introducing pragmatic analysis from natural language processing into this field for the first time, thereby addressing two major limitations of existing research. Our contributions are threefold: (i) establishing a generative reasoning paradigm that improves generalization and provides reference-worthy predictions under cold-start conditions; (ii) defining microenvironment as pragmatic context and injecting the “sequence—microenvironment—function” causal logic into the model; and (iii) constructing the enzyme microenvironment database and developing MEMP to impute missing microenvironment data by jointly learning correlations among parameters.

## 2 Materials and methods

### 2.1 Data collection and overall framework

This work constructs an enzyme microenvironment database containing enzyme sequences, EC numbers, enzymatic reactions, Tems, and pH values. Over 220 000 sequence—EC pairs are sourced from the CLEAN (Yu *et al.* 2023); reactions are crawled from the UniProt (Bairoch *et al.* 2005) and BRENDA (Schomburg *et al.* 2004, Chang *et al.* 2021) databases. Experimental pH and temperature values are available for 60 940 and 3372 enzymes, respectively; the remaining values are imputed by MEMP (Fig.  2a), a multi-task predictor that leverages correlations among pH, temperature, melting temperature, and optimal growth temperature. For MEMP training and evaluation: for pH, the test set comes from EpHod (Gado *et al.* 2025) (*n* = 1971), and the training set integrates EpHod (*n* = 7884) with our database (*n* = 60 940); for temperature, the test set is taken from TOMER (Gado *et al.* 2020) (*n* = 291), and the training set comprises our database (*n* = 3372) plus TOMER (*n* = 2626); for melting temperature and optimal growth temperature, both training (*n* = 6240) and test (*n* = 1550) sets are obtained from DeepTM (Heinzinger *et al.* 2022). For EC prediction, MPA-EC, GraphEC, and CLEAN use the same 220 000 CLEAN pairs for training and evaluate on NEW-392 (392 sequences, 177 ECs) and Price-149 (149 sequences, 100 ECs) (Price *et al.* 2018).

**Figure 2:**
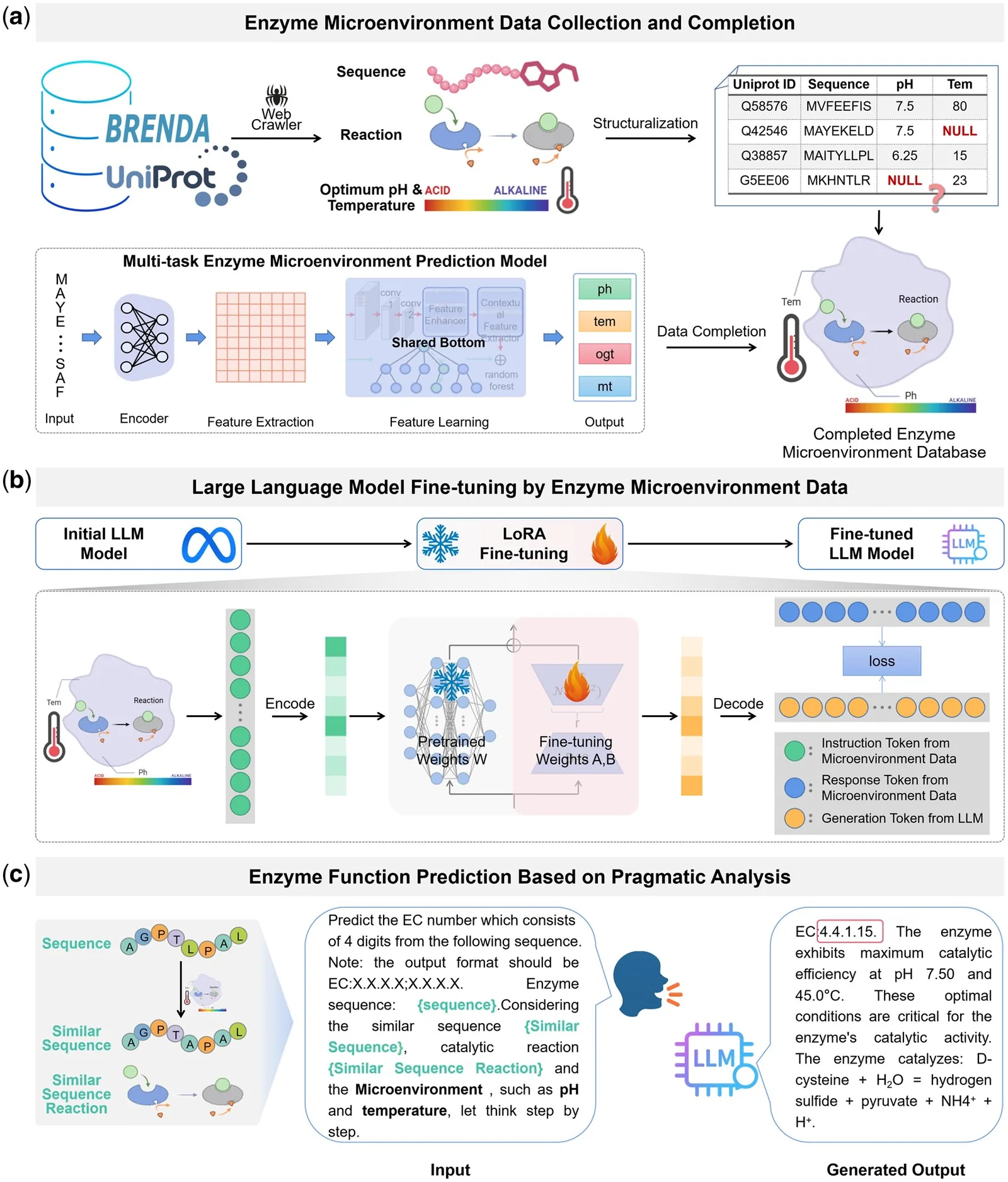
The enzyme microenvironment database and MPA-EC framework. (a) The enzyme microenvironment database construction. This database was built by integrating multiple public resources, where missing experimentally measured pH and temperature values were supplemented by predictions from our MEMP model. (b) Large language model fine-tuning. The query-reasoning chain response training set constructed from the enzyme microenvironment database was used to fine-tune the LLaMA-3.1-8B model via LoRA. The model learnt the chemical causal logic of “sequence—microenvironment—function” by predicting the next token in reasoning chain response. (c) Enzyme function annotation framework, MPA-EC. MPA-EC employs the fine-tuned LLaMA-3.1-8B model to generate EC number predictions with causal reasoning.

Based on the constructed enzyme microenvironment database, we structure and integrate discrete data to establish a fine-tuning dataset for large language model. Then using the LoRA fine-tuning technique, the causal logic of “inferring microenvironment parameters from protein sequences and then reasoning enzyme functions within that microenvironment context” is injected into the LLaMA-3.1-8B pre-trained model (Fig.  2b). The resulting MPA-EC framework (Fig.  2c) uses prompts to guide the fine-tuned model in generating the reasoning process and EC number.

### 2.2 MEMP model architecture

MEMP is a hybrid framework that combines a deep learning module based on a pre-trained language model and a machine learning module based on manual feature engineering, as shown in Fig. 5, available as supplementary data at *Bioinformatics* online. It takes enzyme sequences as input and simultaneously predicts optimum pH (pH), optimum temperature (Tem), melting temperature (Mt), and optimum growth temperature (Ogt).

#### 2.2.1 Deep learning module MEMP-NN architecture

##### 2.2.1.1 Enzyme sequence representation

We employ the pre-trained large language model, LLaMA-3.1-8B, to encode input enzyme sequences, obtaining sequence embeddings $E\in R^{l\times d_{1}}$, where l denotes the sequence length and $d_{1}=4096$ is the dimension size of the pre-trained large language model embeddings. To facilitate batch processing, we apply average pooling along the sequence length dimension to obtain the final fixed-dimensional input representation $X\in R^{B\times d_{1}}$, where B denotes the batch size.

##### 2.2.1.2 Local sequence information learning

We employ a multi-scale convolutional neural network to extract local and multi-resolution sequence information from protein sequences:


(1) bi=σ(Convk= 2i+1(X)), i∈{1,2,3}#

(2) ptlocal=MLPt(σ(Convk=1(Concat(b1,b2,b3))))#

where ${\mathrm{Conv}}^{k=2i+1}(•)$ denotes a convolutional layer with kernel size k, σ(•) is an activation function, and Concat(•) indicates the concatenation operation. Then, we use multi-task multi-layer perceptron (MLP) heads to predict the values for tasks t∈{pH, Tem, Ogt, Mt}.

##### 2.2.1.3 Global sequence information learning

This branch aims to extract global sequence representations from enzyme sequence embeddings through a two-layer feed-forward architecture. Given the input embedding matrix $X\in R^{B\times d_{1}}$, where B denotes the batch size, the module learns the global context information as follows:


(3) H1=σ(LayerNorm(W2·σ(LayerNorm(W1X+b1))+b2))#

(4) ptglobal=σ(LayerNorm(WtH1+bt))#

where $W_{*},\;b_{*}\;$are learnable parameters and LayerNorm(•) is layer normalization operation. Similarly, we construct four task-specific prediction heads to predict the values for tasks t∈{pH, Tem, Ogt, Mt}.

##### 2.2.1.4 Adaptive feature importance learning

We employ channel attention and residual connections to dynamically assess and enhance feature representations. Given the sequence embedding $X\in R^{B\times d_{1}}$ from the pre-trained language model, the module applies four parallel residual blocks with varying dilation rates d∈{1, 2, 4, 8} to capture multi-scale feature interactions. For each block:


(5) Fd=σ(GroupNorm(Convdilation=dk= 1(X))), d∈{1, 2, 4, 8}#

(6) Fr=X+∑d∈{1, 2, 4, 8}Fd#

where σ(•) denotes the activation function, GroupNorm(•) indicates the group normalization operation, and ${\mathrm{Conv}}_{\mathrm{dilation}=d}^{k=\;1}(•)$ is a convolutional layer with kernel size k=1 and dilation rate d. Through residual connections, we integrate these outputs into $F_{r}$. Then, we apply channel attention to dynamically recalibrate feature importance:


(7) Wa=σ(Linear1(σ(Linear2(AdaptiveAvgPool(Frv)))))#

ptadaptive=σ(Linear3(Fr ⨀ Wa))# (8)where AdaptiveAvgPool(•) indicates the global average pooling operation, ⨀ is element-wise multiplication, and Linear(•) denotes the linear layer.

In the deep learning module MEMP-NN, three branches are employed to extract local sequence information, global sequence information, and adaptive feature importance information from enzyme sequences, respectively. These branches output three prediction results $p_{t}^{\mathrm{local}},\;p_{t}^{\mathrm{global}},\;p_{t}^{\mathrm{adaptive}}$ for each task t∈{pH, Tem, Ogt, Mt}. Finally, we apply a nonlinear function to obtain the final deep learning prediction result:


(9) $$\begin{array}{c} \frac{p_{t}^{NN}=W_{t}^{l}\;p_{t}^{\mathrm{local}}+W_{t}^{g}\;p_{t}^{\mathrm{global}}+W_{t}^{a}\;p_{t}^{\mathrm{adaptive}}+}{W_{t}^{f}\;p_{t}^{\mathrm{local}}\;p_{t}^{\mathrm{global}}\;p_{t}^{\mathrm{adaptive}}\;}\;\# \end{array}$$

where $W_{t}^{*}$ are trainable parameters.

#### 2.2.2 Machine learning module MEMP-ML architecture

##### 2.2.2.1 Enzyme feature extraction engineering

We design a 46-dimensional feature vector comprising two components. The first component includes sequence length and the frequencies of the 20 standard amino acids, accounting for 21 dimensions. The second component consists of statistical summaries of five physicochemical properties, namely hydrophobicity, molecular volume, *p*K_*a*_, charge characteristics, and polarity. For each property, we compute the mean, standard deviation, minimum, maximum, and median, yielding 25 dimensions. Together, these features form a feature space that comprehensively captures the physicochemical characteristics $F\in R^{1\times 46}$ of enzyme sequences.

##### 2.2.2.2 Heterogeneous ensemble layer

We adopt an ensemble of five machine learning algorithms including Support Vector Regression (SVR) with RBF kernel, Random Forest (RF) that integrates multiple decision trees, Extra Trees (ET) with stronger randomization, Gradient Boosting (GB) optimized sequentially, and MLP capable of learning enzyme feature information. The predictions from each algorithm are fused via a weighted averaging mechanism. The ensemble prediction $p_{t}^{ML}$ for the task t∈{pH, Tem, Ogt, Mt} is:


(10) ptML=∑i∈{SVR, RF, ET, GB, MLP}Wti pti#

where $W_{t}^{i}$ denotes the learnable weight for model i.

#### 2.2.3 MEMP model training

We obtain the final model prediction by dynamically fusing outputs from the deep learning module and the machine learning module:


(11) y^=α·ptNN+(1-α)·ptML#

where the fusion weight α is adaptively adjusted based on the performance of each pathway on the validation set. This fusion strategy ensures optimal balance across different prediction tasks.

##### 2.2.3.1 Multi-task loss function

MEMP employs a multi-task loss defined as the weighted sum of individual task losses, with weights adjusted according to prediction difficulty, value ranges, and biological importance. For pH prediction, we use a composite loss combining regression and classification objectives, including a weighted regression term, a boundary constraint (pH 0–14), MSE, and an auxiliary classification loss. For temperature, Ogt, and Mt, we adopt Smooth L1 loss for robustness against outliers.

##### 2.2.3.2 MEMP optimization strategy

Training follows a two-phase regimen: the deep learning modules are pre-trained independently to establish robust representations, followed by joint optimization of all components through combined loss backpropagation.

### 2.3 Fine-tuning large model

To inject the causal logic of inferring microenvironment parameters from protein sequences and then reasoning enzyme functions, this study employs LoRA fine-tuning strategy. Specifically, we freeze all original parameters of the pre-trained LLaMA-3.1-8B model and introduce trainable rank-decomposition matrices into the self-attention and feed-forward modules. Using the LoRA fine-tuning strategy enables adaptation to downstream tasks with minimal additional parameters while preserving the rich knowledge acquired during pre-training.

For data preparation, we construct an instruction-tuning dataset for enzyme functional annotation. Each training sample comprises two components: an inquiry regarding the EC number of an enzyme sequence, and a corresponding response presenting a step-by-step reasoning chain. The chain first infers the enzyme’s microenvironment characteristics from the sequence, such as pH and temperature, and then utilizes these characteristics to deduce the functional classification. The reasoning chain incorporates microenvironment data retrieved from the enzyme microenvironment database. By fine-tuning the model to predict the next token in the reasoning response, we instill the above causal logic into the pre-trained large language model.

### 2.4 Enzyme EC number annotation

This study proposes the MPA-EC framework, re-framing sequence-function annotation as a text generation task. Differing from traditional discriminative models, this framework dynamically generates text outputs containing complete causal reasoning chains based on input enzyme amino acid sequences, thereby parsing the corresponding EC numbers.

The generative large language model utilizes the LLaMA-3.1-8B model as the initial model and is fine-tuned with instructions from the enzyme microenvironment database. This work employs the above model as the core inference engine, guiding it through step-by-step reasoning by constructing prompts that include the sequence, similar enzyme sequences, and similar enzyme catalytic reactions. Specifically, we use a batch-efficient sequence similarity search module based on MMSeq2 to rapidly locate the most similar sequences for query sequences from the enzyme microenvironment database. Notably, to ensure validation accuracy and fairness, we remove sequences identical to the query sequence from the enzyme microenvironment database. Similar enzyme catalytic reactions are also sourced from this database and structurally integrated into the prompt engineering. Ultimately, the model generates predictions following an “input—think—output” instruction format. Standardized EC numbers are extracted through post-processing that regularizes the output text.

## 3 Results

### 3.1 Enzyme EC number annotation

This study proposes the Microenvironment Awareness Pragmatic Analysis model MPA-EC for generative enzyme function reasoning and validates its annotation performance on two public datasets: NEW-392 and Price-149.

We compare MPA-EC with five state-of-the-art EC number predictors: ECPred (Dalkiran *et al.* 2018), CLEAN (Yu *et al.* 2023), DeepEC (Ryu *et al.* 2019), ProteInfer (Sanderson *et al.* 2023), and GraphEC (Song *et al.* 2024), showing the results in Fig. 3a and Fig. 4a, available as supplementary data at *Bioinformatics* online. Compared with the other methods, CLEAN, GraphEC, and MPA-EC show substantial improvements, benefiting from the more comprehensive training dataset curated by CLEAN. On the Price-149 dataset, MPA-EC outperforms all compared models, achieving an F1 score of 0.7037. Compared to the suboptimal model GraphEC, MPA-EC improves precision, recall, and F1 score by 0.0400, 0.0654, and 0.0906, respectively. On the NEW-392 dataset, which covers a broader range of EC categories, MPA-EC achieves an F1 score of 0.7112, appreciably higher by 0.1068 than that of GraphEC (Song *et al.* 2024) (0.6044). The recall reaches 0.6686, indicating that MPA-EC comprehensively covers various enzyme functional labels in the test set while maintaining the accuracy of the generated results.

**Figure 3:**
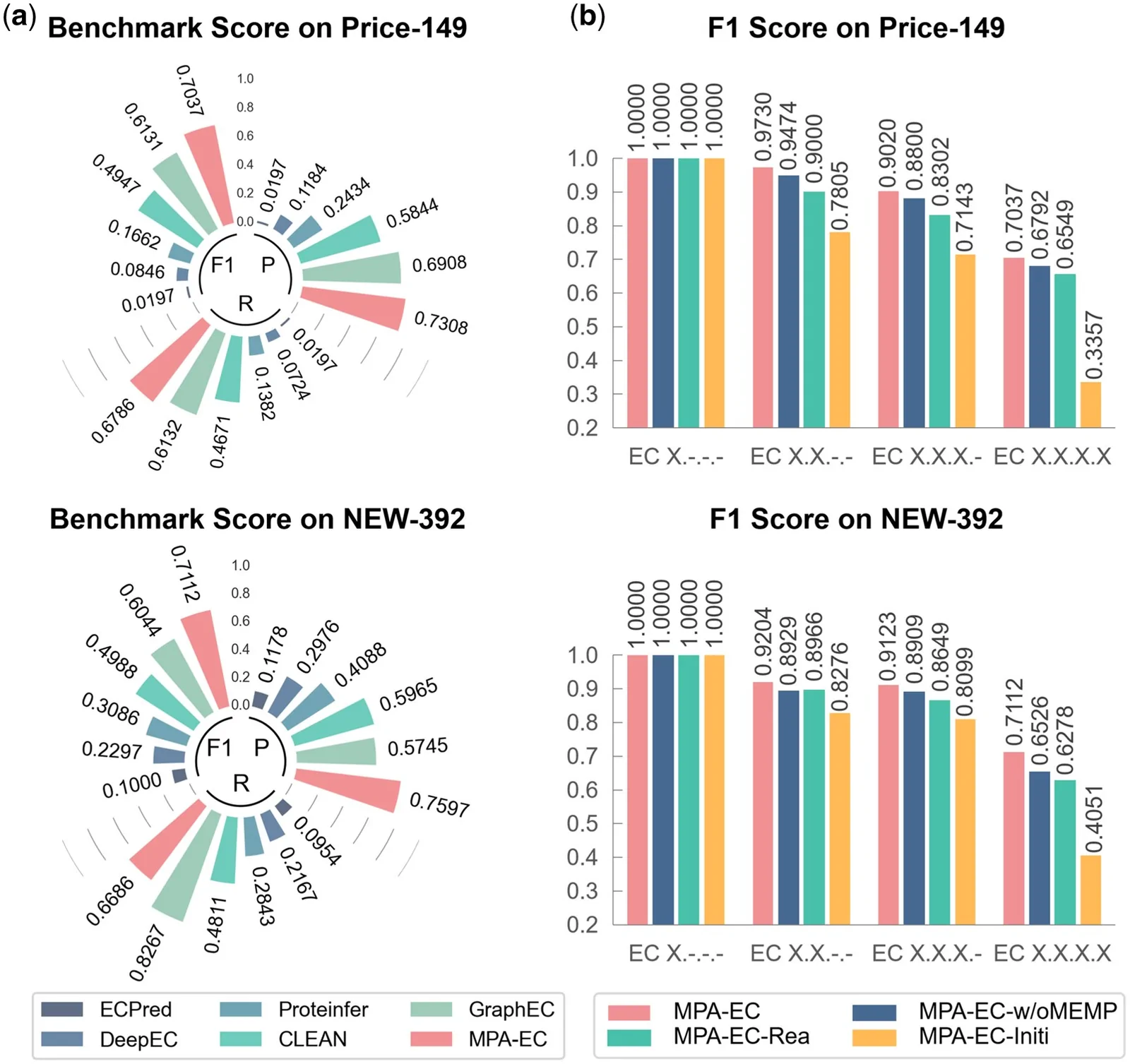
The enzyme EC number annotation performance. (a) Performance comparison with five state-of-the-art models on the Price-149 and NEW-392 datasets evaluated by precision, recall, and F1 score. (b) Ablation experiments focusing on various information for fine-tuning the large language model.

**Figure 4:**
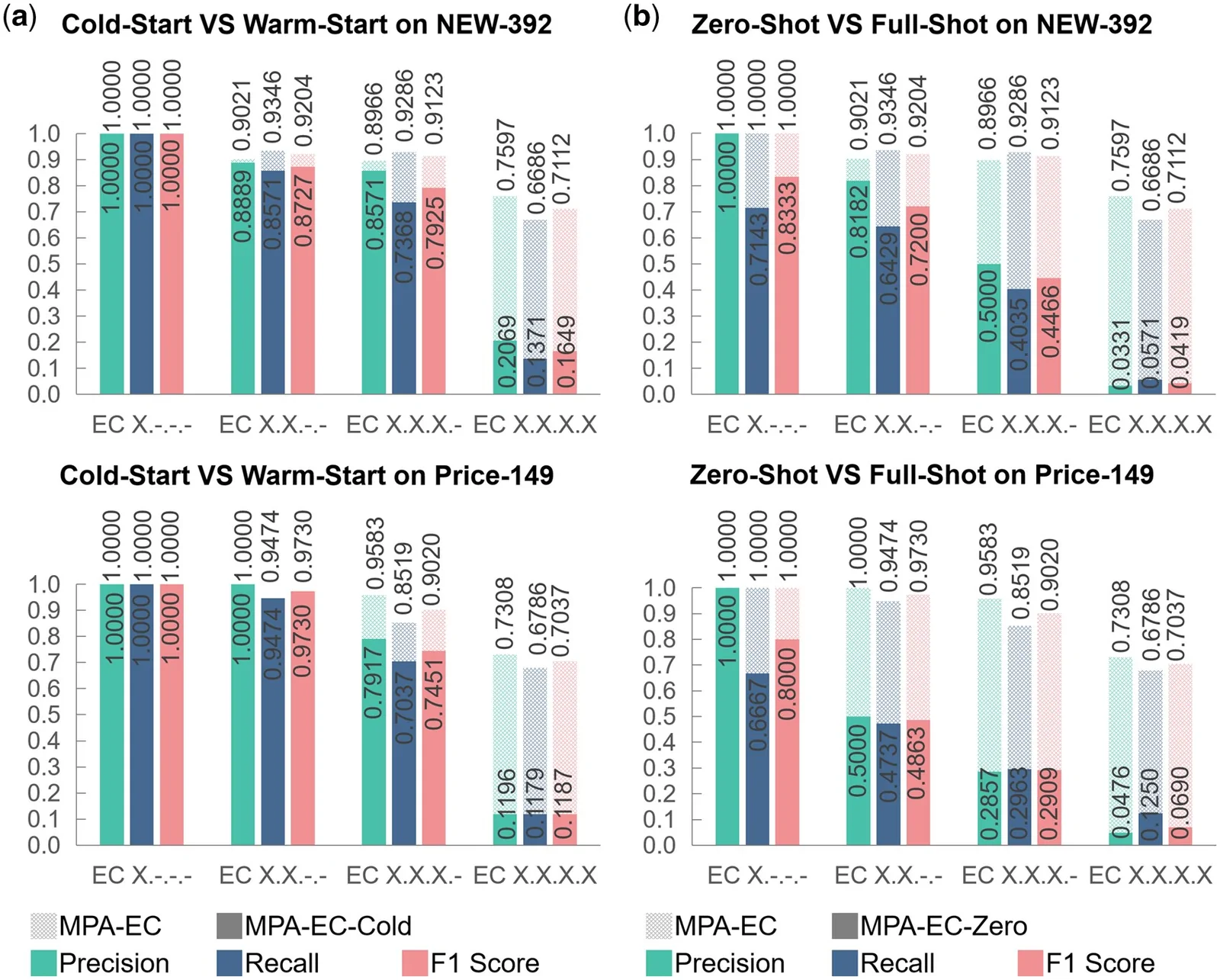
Enzyme function reasoning in cold-start and zero-shot conditions. (a) Analysis under cold-start condition. MPA-EC-Cold excludes test-set EC labels from training to assess generalization to unseen activities. (b) Analysis under zero-shot condition. MPA-EC-Zero relies solely on pre-training knowledge without domain-specific fine-tuning. The comparison highlights the value of fine-tuning for reliable EC prediction.

To isolate the contribution of generative reasoning from the input information itself, we designed a discriminative DNN baseline that receives exactly the same structured input as MPA-EC, including sequence embeddings, Top-1 similar sequence embeddings with their EC labels, and reaction features, with the sole difference being discriminative classification versus generative reasoning, as shown in Tables 6 and 7, available as supplementary data at *Bioinformatics* online. For reference, a DNN using only sequence features achieves Level-4 F1 scores of 0.1101 on Price-149 and 0.1604 on NEW-392. With the full input containing retrieved sequences, EC labels, and reaction features, the DNN improves only marginally to 0.1131 and 0.1741, respectively, far below MPA-EC’s 0.7037 and 0.7112. These results suggest that while additional information provides some benefit, how that information is utilized matters more, and MPA-EC’s performance advantage is largely attributed to its generative reasoning capability rather than information access alone.

We further performed head-to-head McNemar tests between MPA-EC and CLEAN on the ECall set; all 10 runs yielded *p* < 1*e^−^*^10^, confirming that the performance advantage is statistically significant (Figs 2 and 3, available as supplementary data at *Bioinformatics* online). Different from discriminative approaches that rely on contrastive learning and graph structures, MPA-EC’s generative reasoning framework simulates the causal logic chain of “sequence—microenvironment—function.” This approach not only achieves optimal performance but also generates interpretable reasoning trajectories, providing text-based causal arguments to advance enzyme function understanding.

### 3.2 Large language model fine-tuning

To train the large language model to learn the causal logic chain of “inferring microenvironment parameters from protein sequences and then reasoning enzyme functions within that microenvironment context,” we employ LoRA to fine-tune LLaMA-3.1-8B on question–answer samples from the enzyme microenvironment database.

The experimental results, shown in Fig. 3b, demonstrate that annotation performance improves with enriched fine-tuning information. MPA-EC-Init is fine-tuned with EC labels only, achieving an F1 of 1.0000 at the mainclass level but dropping sharply at deeper levels (0.7805, 0.7143, and 0.3357 on Price-149). This indicates that learning the output format alone, without understanding the underlying chemical logic, is insufficient for accurate annotation. MPA-EC-Rea incorporates reaction information and also learns mappings from highly similar retrieved EC labels to the true ones, enabling the model to learn deeper “sequence—reaction—function” associations. On Price-149, its Level-4 F1 rises from 0.3357 to 0.6549, and a similar trend is observed on NEW-392 (from 0.4051 to 0.6278). The full MPA-EC further integrates microenvironment data, achieving the best overall performance with a Level-4 F1 of 0.7037 on Price-149, a 0.0488 improvement over MPA-EC-Rea. These results suggest that microenvironment parameters provide contextual constraints that guide the model toward more biochemically relevant reasoning.

To quantify the impact of imputed microenvironment values, we further compare MPA-EC against MPA-EC-w/oMEMP, which uses only experimental pH/temperature values (with MEMP-predicted samples replaced by microenvironment-free samples). On Price-149, the Level-4 F1 values are 0.7037 for MPA-EC, 0.6792 for MPA-EC-w/oMEMP, and 0.6549 for MPA-EC-Rea. MPA-EC consistently outperforms MPA-EC-w/oMEMP, indicating that despite the noise in MEMP predictions, the broader training coverage from imputed data provides useful signals. MPA-EC-w/oMEMP in turn outperforms MPA-EC-Rea, confirming that even partial experimental microenvironment information is beneficial. A similar trend is observed on NEW-392. These results suggest that while current imputation accuracy is limited, the statistical correlations captured by MEMP still serve as valuable constraints, and further improvements are expected as more experimental data accumulate.

### 3.3 Enzyme function reasoning in the cold-start

We validate MPA-EC-Cold under cold-start conditions on Price-149 and NEW-392, as shown in Fig.  4a, where all test-set EC labels are removed from training and the microenvironment database to simulate annotation of previously unseen enzymes. The model achieves strong performance at mainclass and subclass levels (e.g. F1 of 1.0000 and 0.9730 on NEW-392), with only a small drop from the warm-start setting at the subclass level (0.0477 on Price-149). At the subsubclass level, F1 scores remain reasonable at 0.7925 and 0.7451 on the two datasets. However, performance declines substantially at the designation level, where F1 drops by 0.5463 and 0.5850 on NEW-392 and Price-149, respectively. This is expected, as distinguishing closely related isoenzymes requires highly specific knowledge that is difficult to infer without prior exposure. In contrast, the small gaps at the upper three levels between cold-start and warm-start conditions confirm that the model’s reasoning relies on learned causal logic rather than memorization. Overall, MPA-EC provides reliable and reference-worthy hierarchical predictions at the mainclass, subclass, and subsubclass levels for novel enzymes.

### 3.4 Enzyme function reasoning in the zero-shot conditions

In the zero-shot setting, we evaluate the base LLaMA-3.1-8B model without any enzyme-related fine-tuning (MPA-EC-Zero) and compare it against the fine-tuned MPA-EC. As shown in Fig. 4b, the performance gap between the two models widens progressively across EC levels. On NEW-392, MPA-EC-Zero achieves an F1 of 0.8333 at the mainclass level versus 1.0000 for MPA-EC, and the gap expands to 0.4657 at the subsubclass level and 0.6693 at the designation level. A similar trend is observed on Price-149, where performance drops from 0.8000 to 0.0690 at the designation level. Notably, even with highly similar sequences and their EC labels included in the prompt, the zero-shot model still performs poorly, as it has not learned the mapping from sequence features to functional labels. These results indicate that while the base model can perform coarse-grained annotation using prior knowledge from pre-trained corpora, it fails at fine-grained reasoning that requires understanding of biochemical contexts such as pH and temperature. The progressively widening gap underscores the necessity of injecting the “sequence—microenvironment—function” reasoning logic via fine-tuning to enable reliable and detailed EC prediction.

### 3.5 Analysis of representative failure cases

To better understand the limitations of MPA-EC, we analyzed three representative misclassification cases from the test set, as summarized in Table 1.

**Table 1:** Representative misclassification cases of MPA-EC on the test set.

Error type	Uniprot ID	True label	Prediction label	Similar sequence label
**Case1**	A0A075HNX4	1.1.3.20	1.1.3.37	1.1.3.37
**Case2**	G5ECS3	5.4.99.9	2.1.1.173	–
**Case3**	F2XF97	4.2.3.104	4.2.3.71	4.2.3.44

#### 3.5.1 Case 1: misled by similar sequence

For sequence A0A075HNX4 (true EC 1.1.3.20), the top hit retrieved by MMseqs2 was Q6CG88 (38.6% sequence identity), which has EC 1.1.3.37. In contrast, Q9LW56 and B5WWZ8, which share the same EC 1.1.3.20 as the query, showed only 28.8% and 29.2% sequence identity to the query, respectively, and were ranked behind the top hit. This suggests that MPA-EC can be misled when the most similar sequence carries a different EC label, especially when the truly same-EC homologs are more distantly related.

#### 3.5.2 Case 2: lack of similar sequences

For G5ECS3 (true EC 5.4.99.9), MMseqs2 did not retrieve any sequence with significant similarity. In the absence of reliable retrieval clues, the model tended to favor EC 2 (a more frequent class in the training set) over the less abundant EC 5, resulting in a misclassification (2.1.1.173).

#### 3.5.3 Case 3: reasoning divergence from retrieval

For F2XF97 (true EC 4.2.3.104), MPA-EC predicted 4.2.3.71, while the top similar sequence has EC 4.2.3.44. The prediction differs from the retrieved label, confirming that MPA-EC does not simply copy the nearest neighbor’s annotation. Nevertheless, the reasoning process can still produce errors at the finest EC level.

These cases suggest that MPA-EC’s primary failure modes are (i) being misled by functionally distinct but sequence-similar homologs and (ii) lacking sufficient retrieval support for orphan sequences. Encouragingly, the model does not blindly copy retrieval labels, confirming that its predictions arise from a reasoning process rather than simple memorization or retrieval replication. Further improvements may be achieved by incorporating more robust homology filtering and enhancing the model’s ability to handle low-homology sequences.

### 3.6 Results of enzyme microenvironment prediction

To complete missing microenvironment data and capture correlations among parameters, we propose MEMP, a multi-task framework with a hybrid architecture combining deep learning (MEMP_NN) and machine learning (MEMP_ML). As shown in Fig. 5, available as supplementary data at *Bioinformatics* online, MEMP takes enzyme sequences as input and predicts pH, Tem, Mt, and Ogt.

**Figure 5:**
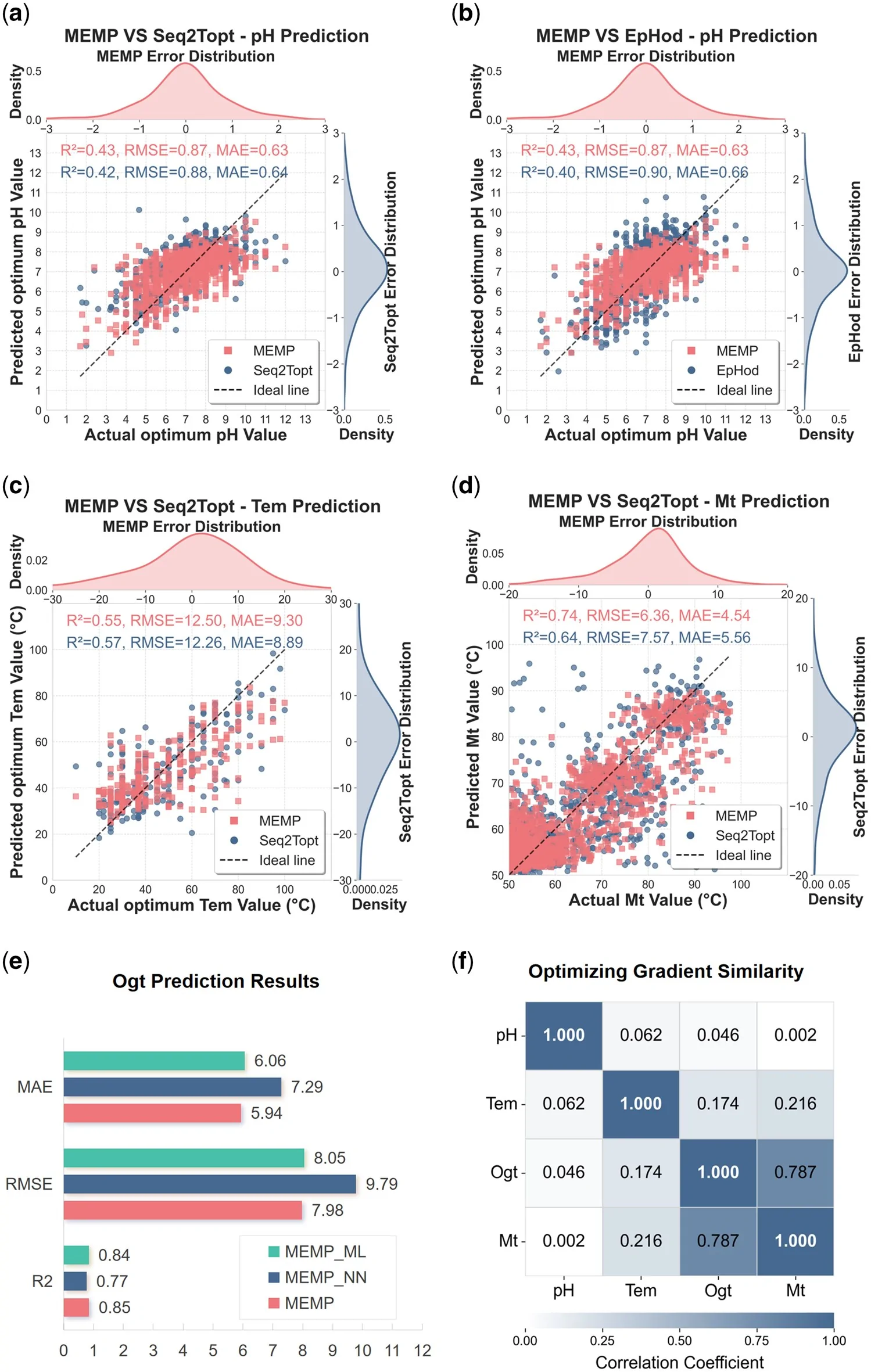
Enzyme microenvironment parameter prediction. (a and b) Prediction results comparison for enzyme pH. (c) Prediction results comparison for enzyme Tem. (d) Prediction results comparison for Mt. (e) Architecture analysis of MEMP for Ogt prediction, showing the performance of its neural network-based module (MEMP_NN), its machine learning-based module (MEMP_ML), and the integrated full model (MEMP). (f) Gradient similarity heatmap across different microenvironment prediction tasks during optimization.

To validate the performance of MEMP, we perform a systematic evaluation and comparison on four public datasets. For pH prediction, as shown in Fig. 5a and b, MEMP (R2 = 0.43, RMSE = 0.87, and MAE = 0.63) outperforms the baseline model Seq2Topt (Qiu *et al.* 2025) (R2 = 0.42, RMSE = 0.88, and MAE = 0.64) and the EpHod (Gado *et al.* 2025) model (R2 = 0.40, RMSE = 0.90, and MAE = 0.66), demonstrating its stable and improved prediction capability. For the Tem prediction task, on the TOMER (Gado *et al.* 2020) test set (*n* = 291) shown in Fig. 5c, MEMP achieves competitive results (R2 = 0.55, RMSE = 12.50, and MAE = 9.30), comparable to the current state-of-the-art Seq2Topt (Qiu *et al.* 2025) model (R2 = 0.57, RMSE = 12.26, and MAE = 8.89). In the Mt prediction task, as shown in Fig. 5d, MEMP achieves a prediction result of R2 = 0.74, RMSE = 6.36, and MAE = 4.54, comprehensively outperforming the baseline models Seq2Topt R2 = 0.64, RMSE = 7.57, and MAE = 5.56. The error density plot shows a more concentrated error distribution, indicating higher prediction stability. For the Ogt prediction task, Fig. 5e demonstrates that the complete MEMP model, which integrates deep representation learning and manual feature engineering (RMSE = 5.94), outperforms its independent submodules MEMP_NN (RMSE = 7.29) and MEMP_ML (RMSE = 8.05). This validates the effectiveness of the hybrid architecture.

During the optimization process, we further analyze the intrinsic connections among different microenvironment parameters by visualizing the optimization gradient similarity heatmap of trainable model parameters, as shown in Fig. 5f. Gradient similarity analysis reveals consistently positive correlations among tasks, indicating that microenvironment parameters are biologically interrelated. MEMP leverages these correlations via its shared representation layer, achieving competitive performance across tasks compared to single-task specialists such as EpHod (Gado *et al.* 2025) and Seq2Topt (Qiu *et al.* 2025).

## 4 Conclusion

This study presents a computational framework that integrates microenvironment information with generative reasoning for enzyme function annotation. Our contributions are threefold. First, we establish a generative reasoning paradigm based on large language models that improves generalization to emerging enzymatic activities and provides reference-worthy predictions under cold-start conditions. Second, we define microenvironment as pragmatic context and inject the explicit “sequence–microenvironment–function” causal logic into the model, enabling interpretable, context-aware annotation. Third, we construct an enzyme microenvironment database and develop MEMP, a multi-task framework that imputes missing microenvironment data by jointly learning correlations among parameters.

Experimental results demonstrate that MPA-EC significantly improves annotation accuracy, achieving a 0.1068 increase in F1 score over the state-of-the-art, and generates reference-worthy functional labels for enzymes under cold-start conditions. This work marks a transition from discriminative classification reliant on known labels to generative reasoning grounded in biochemical context.

## Supplementary Material

btag769_Supplementary_Data
