# Supplementary material for: Microenvironment awareness pragmatic analysis for generative enzyme function reasoning
Source: Bioinformatics. 2026 Oct 13;42(11):btag769. doi: 10.1093/bioinformatics/btag769 (PMC13728851; doi:10.1093/bioinformatics/btag769)
Supplement: Supplementary file 1 [file btag769_supplementary_data.zip › Supplementary Information.pdf]

Supplementary Information

Paper Title: Microenvironment Awareness Pragmatic Analysis for Generative Enzyme Function Reasoning

Table of Contents

Supplementary Figures

1 Multi-trial performance for enzyme function annotation.
2 McNemar test results between MPA-EC and CLEAN on the ECall test set (n=541).
3 Level-4 F1 score comparison between MPA-EC (10 independent runs) and CLEAN (deterministic) on the ECall test set.
4 The enzyme EC number annotation performance.
5 Multitask enzyme microenvironment prediction Framework MEMP.
6 Case study of model reasoning in cold-start and zero-shot conditions.

Supplementary Tables

1 Sample data for fine-tuning.
2 Hyperparameter space of fine-tuning process.
3 Hyperparameter space of enzyme microenvironment prediction model.
4 Performance comparison of DNN baseline models on the Price-149 dataset.
5 Performance comparison of DNN baseline models on the NEW-392 dataset.
6 Performance comparison of DNN baseline models (sequence-only, full prompt, and without natural language description) on the Price-149 dataset.
7 Performance comparison of DNN baseline models (sequence-only, full prompt, and without natural language description) on the NEW-392 dataset.
8 Performance comparison of MPA-EC under different sequence similarity thresholds on the Price-149 dataset.
9 Performance comparison of MPA-EC under different sequence similarity thresholds on the NEW-392 dataset.
10 Comparison between the Retrieval Only baseline and MPA-EC across EC levels.
11 Progressive matching score and confidence score analysis for enzyme EC number annotation.

Supplementary Algorithms

1 Progressive matching score calculation for evaluating enzyme functional annotation performance.
2 Confidence score calculation for evaluating enzyme functional annotation performance.

Multi-trial performance for enzyme function annotation

To assess the robustness of MPA-EC, we conducted ten independent validation runs on the combined test set (NEW-392 and Price-149) and reported the mean, standard deviation, and 95% confidence intervals for performance metrics at each EC level, as detailed in **Supplementary Fig. 1**. The model shows consistently small standard deviations across all levels: at Level 2, the mean F1 is 0.9489 (±0.0058) with a 95% confidence interval of [0.9449, 0.9525]; at Level 3, the mean F1 is 0.9240 (±0.0070) with an interval of [0.9186, 0.9278]; and at Level 4, the mean F1 is 0.7071 (±0.0074) with an interval of [0.7029, 0.7125]. Furthermore, pairwise McNemar's tests on the ten runs yielded p-values all greater than 0.05 (minimum p = 0.099), confirming no significant difference in predictions across runs. These results demonstrate that MPA-EC produces highly consistent and reproducible functional annotations across all EC levels, with little random variation, making it suitable for practical enzyme function annotation tasks. For subgroups with limited sample sizes (e.g., DS6: n=14, DS4.1: n=7, DS7: n=4), we explicitly indicate the sample size in the main text and present these results only as descriptive trends.

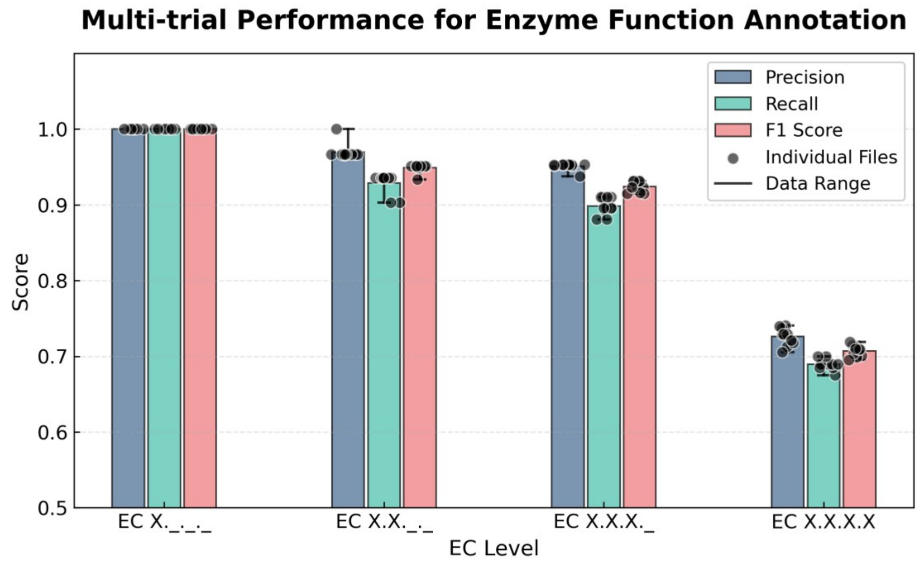

Supplementary Fig. 1: Multi-trial performance for enzyme function annotation. Performance metrics (precision, recall, F1 score) were evaluated through ten independent validation runs on the combined dataset comprising NEW-392 and Price-149 samples.

To statistically support the comparative claim of MPA-EC's superiority, we performed McNemar's tests between MPA-EC and CLEAN on the identical ECall test set (n=541). GraphEC was not included in this analysis due to difficulties in fair end-to-end reproduction. For each of MPA-EC's 10 independent runs, we paired the Level-4 predictions against CLEAN's single deterministic prediction on a per-sample basis and performed McNemar's test with continuity correction. As shown in **Supplementary Fig. 2**, all 10 runs yielded $p < 1e-10$.

To provide a single robust MPA-EC prediction, we applied majority voting across the 10 runs. As shown in **Supplementary Fig. 3**, MPA-EC (majority vote) achieved a Level-4 F1 of 0.7078, compared to CLEAN's 0.4443, with a McNemar p value of 9.33e-15. Level-specific McNemar tests further showed that MPA-EC significantly outperforms CLEAN at L1, L2, and L3 levels ($p < 0.05$ for all). In summary, the paired McNemar tests directly compare MPA-EC and CLEAN on identical samples, with

all 10 runs and the majority-vote aggregation yielding p < 1e-10, confirming that the performance advantage of MPA-EC is statistically supported.

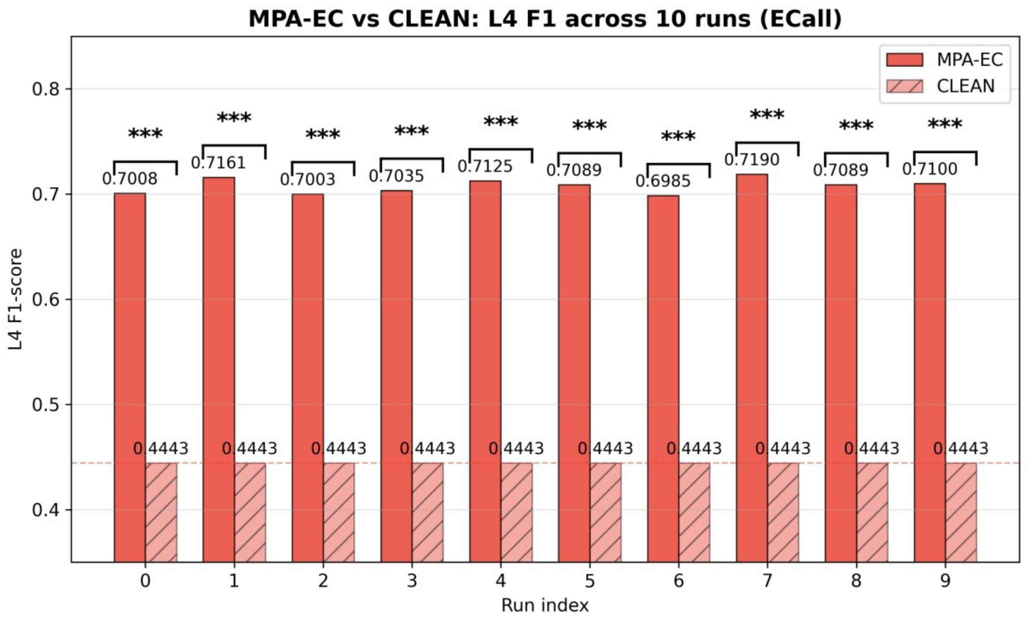

Supplementary Fig. 2: McNemar test results between MPA-EC and CLEAN on the ECall test set (n=541).

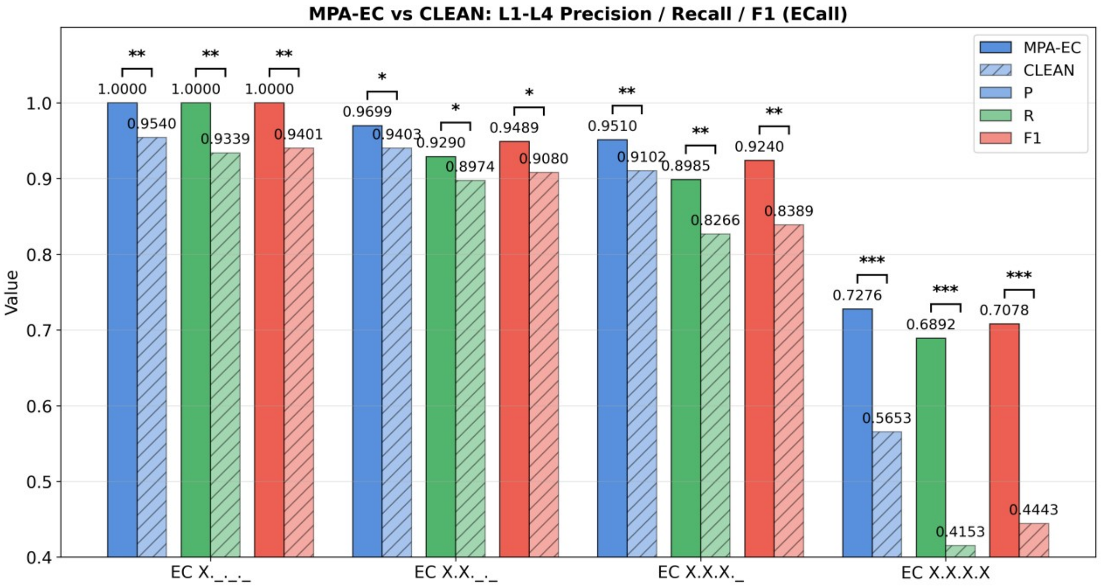

Supplementary Fig. 3: Level-4 F1 score comparison between MPA-EC (10 independent runs) and CLEAN (deterministic) on the ECall test set.

EC Annotation performance evaluation

To comprehensively evaluate the enzyme functional annotation performance of MPA-EC, this study introduces two new metrics beyond precision, recall, and F1 score: progressive matching score and confidence score, as detailed in **Supplementary Algorithms. 1** and **2**. The progressive matching score is designed to quantify the depth of alignment between the generated output and the ground-truth EC number along the hierarchical classification levels. The hierarchical structure of EC numbers (format EC X.X.X.X) inherently reflects a progression in biological specificity: a match at a higher level (e.g., the mainclass and the subclass level) generally indicates broader functional similarity and evolutionary conservation compared to a match at a lower level. Therefore, the score employs a weighted decay mechanism, applying decreasing weights to match results across four EC levels (weights: 1.0, 0.7, 0.49, 0.343; decay factor: 0.7). The progressive matching score is calculated by cumulatively weighting the scores from each matching layer and is normalized to the [0,1] range, where a higher value indicates that the generated result aligns with the true function at a more specific hierarchical level.

The confidence score evaluates the self-awareness of the model regarding the reliability of its generated results. This score is based on repeated independent generations using identical inputs and integrates two core consistency metrics. First, complete consistency ($C_{consistency}$, weight 40%) is defined as the proportion of the most frequently generated EC number, reflecting the concentration of the model's most certain conclusion. Second, average pairwise similarity ($C_{similarity}$, weight 60%) is computed by averaging the similarity across all pairs of generated results, where the similarity between two results is the count of matching digit positions divided by four, thereby indicating overall output agreement.

We combine the Price-149 and NEW-392 datasets for a more granular evaluation to further explore the correlation between MPA-EC's generative capability and the characteristics of major enzyme classes, As shown in **Supplementary Figs. 4 (b)** and **(c)**.

MPA-EC achieves high matching scores for DS2 (transferases, progressive matching score 0.938) and DS6 (ligases, score 0.938), while also demonstrating solid performance for DS1 (oxidoreductases, score 0.891) and its subcategory DS1.14 (score 0.911). The model's confidence scores for these classes (DS2: 0.926, DS1: 0.871, DS1.14: 0.910, DS6: 0.955) show good alignment with the progressive matching scores, indicating a reasonable capacity for self-assessment of its output quality. The catalytic processes in these better-performing categories typically involve well-defined core biochemical events, such as substrate transfer, electron transfer, or bond formation. The realization of these functions is often directly linked to enzyme stability and activity under specific physicochemical conditions like pH and temperature. Therefore, the "sequence - microenvironment - function" inference framework adopted by MPA-EC proves effective for modeling and generating annotations for such enzymes.

The performance in DS3 (hydrolases, progressive matching score 0.667) objectively reflects the biological complexity of this enzyme class. Although microenvironment parameters serve as useful contextual clues, the diverse catalytic mechanisms and high functional redundancy of hydrolases result in reduced annotation performance. Notably, the confidence score generated by MPA-EC for this class (0.710) is correspondingly conservative relative to its matching score, demonstrating the model's capacity for nuanced self-assessment regarding output quality. For DS5 (isomerases, progressive matching score 0.945, sample size = 17) and DS7 (translocases, progressive matching score 0.932, sample size = 4), the relatively lower confidence scores (0.686 and 0.674, respectively) despite high matching scores can be attributed to the model's cautious self-assessment in low-sample scenarios. While it achieves accuracy on the available samples, the limited number of training examples for these

enzyme classes likely increases the model's uncertainty, leading it to assign more conservative confidence estimates. This behavior demonstrates a sophisticated self-regulation mechanism in which the model appropriately down-weights its certainty when statistical support is limited, preventing overconfident predictions in data-sparse regions.

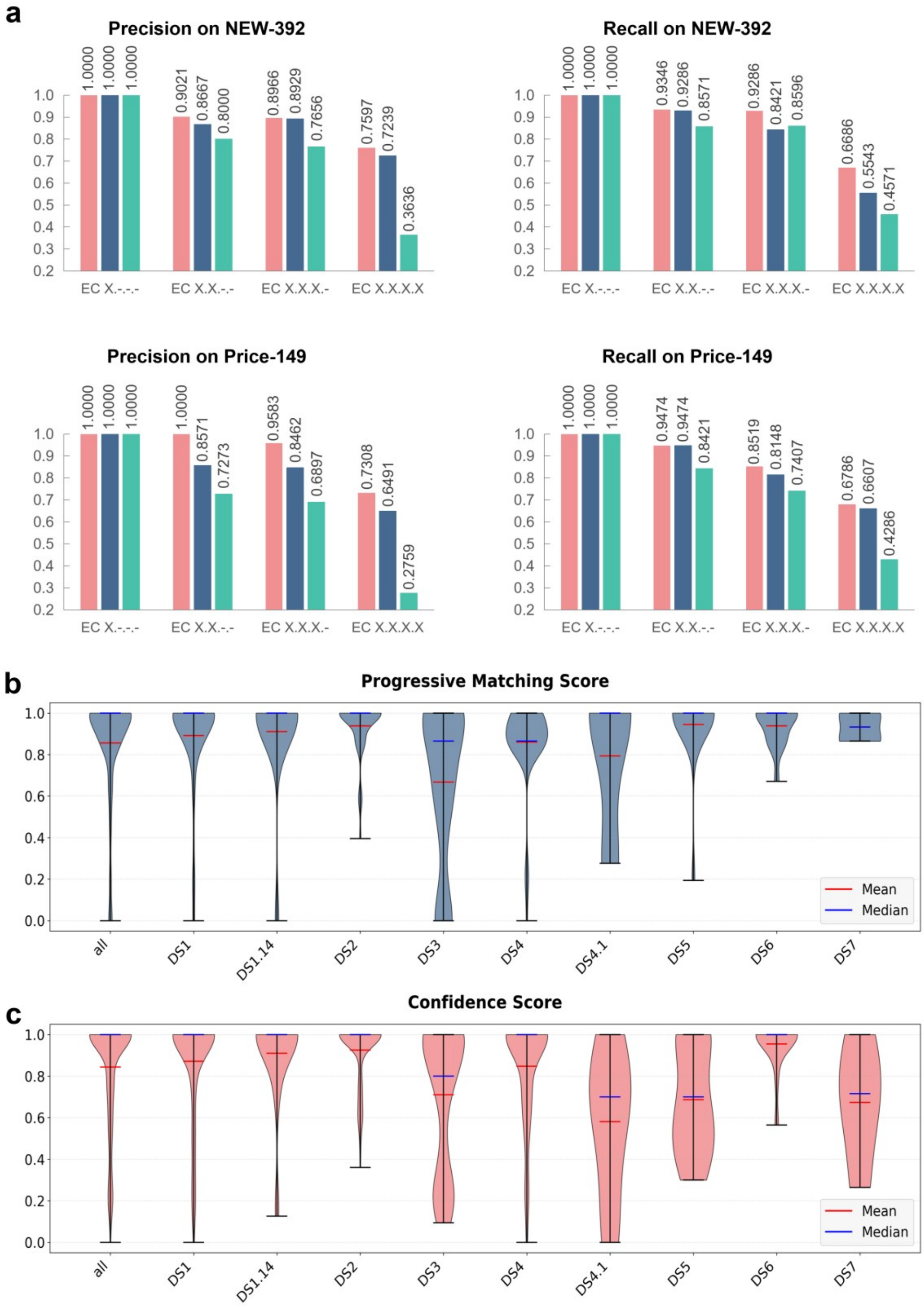

Supplementary Fig. 4: The enzyme EC number annotation performance. a, PAblation experiments focusing on various information for fine-tuning large language model. **b, c,** Progressive matching score and confidence score analysis for enzyme EC number annotation. The “all” group represents the overall performance across all test samples including the Price-149 and NEW-392 datasets, “DS” group denotes the performance of sample groups categorized by EC number major or minor classes. For example, “DS4.1” represents all samples belonging to EC 4.1.X.X (carbon-carbon cleavage enzymes).

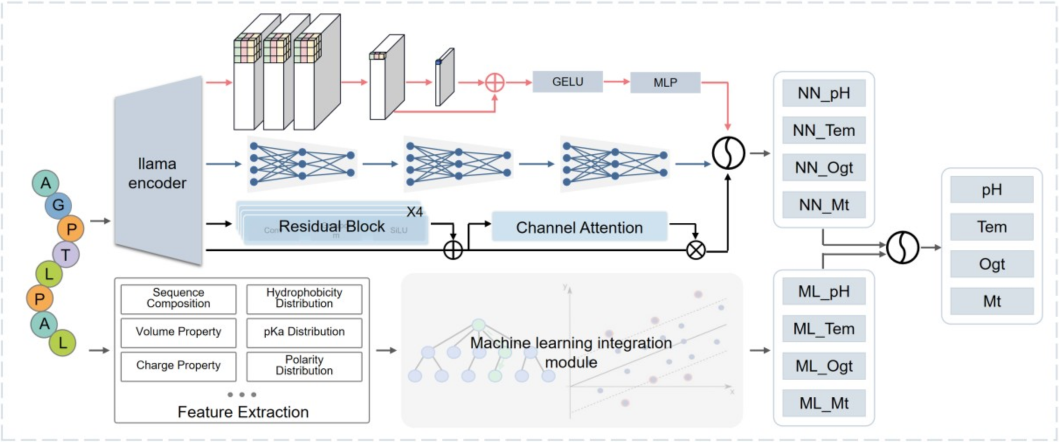

Supplementary Fig. 5: Multitask enzyme microenvironment prediction Framework MEMP.

Case study of model reasoning in the cold-start and the zero-shot conditions

To directly compare the reasoning performance of models under cold-start (MPA-EC-Cold), zero-shot (MPA-EC-Zero), and full fine-tuning (MPA-EC) conditions, this study presents two specific enzyme function annotation cases, as shown in **Supplementary Fig. 6**.

For o-succinylbenzoate synthase, which catalyzes hydrolysis under the microenvironment of pH 8.0 and 55°C, MPA-EC accurately generates the EC number (4.2.1.113), matches the microenvironment of pH 8.00 and 60.0°C, and produces the correct reaction equation, demonstrating its ability to perform accurate inference. MPA-EC-Cold generates an EC number of 4.2.1.55, whose designation level number is incorrect, but it accurately outputs the mainclass, subclass, and subsubclass level labels. The generated microenvironment parameters, pH 7.50 and 98.0°C, show consistent trends with the actual values, both indicating a weakly alkaline environment. Although it provides the erroneous reaction equation "(R)-2-methyl-3-hydroxybutanoyl-CoA = (R)-2-methylbut-2-enoyl-CoA + H2O," it maintains logical consistency with the reaction type (hydrolytic cleavage) and the predicted EC number (4.2.1.55, also a hydrolase). This indicates that the model has learned causal reasoning logic through fine-tuning and can generate reference-worthy function labels even without prior knowledge of the enzyme. In contrast, MPA-EC-Zero incorrectly labels the enzyme as DNA polymerase (EC 2.7.2.3; 2.7.2.5) with output pH and temperature ranges of 7.0-8.0 and around 37°C, which deviate appreciably from the actual values. Moreover, the proposed reaction mechanism, irrelevant to the actual reaction equation, indicates its inability to perform effective reasoning.

For the redox reaction catalyzed by D-2-hydroxyglutarate dehydrogenase under a microenvironment of pH 7.4 and 37°C, similarly, MPA-EC accurately infers the enzyme function as EC 1.1.99.39. MPA-EC-Cold reasons EC 1.1.99.40; 1.1.2.4. Although the designation level number is incorrect, it correctly identifies the redox enzyme. The generated microenvironment (pH 8.50, 85.0°C) shows some deviation from the actual value but still reflects a reasonable inference of weakly alkaline characteristics. Its erroneous reaction output (lactate dehydrogenase) aligns with its predicted redox enzyme category, further demonstrating logical consistency and the ability to generate reference-worthy labels. The MPA-EC-Zero model mistakes it as a kinase (EC 2.7.1.2; 2.7.1.23) and generates an irrelevant phosphogroup transfer description.

The above cases more clearly demonstrate that the generative large language model based on Microenvironment Awareness Pragmatic Analysis provides unique value in enzyme function annotation tasks. Differing from discriminative models, MPA-EC generates reference-worthy EC number labels and reasonable physicochemical property inferences for novel enzymes under the cold-start condition, while maintaining internal logical consistency in its outputs. In contrast, the MPA-EC-Zero model without fine-tuning fails to effectively reason with domain-specific information under the zero-shot condition. This comparison demonstrates that fine-tuning to inject the causal logic of "sequence - microenvironment - function" enables the large language model to acquire reliable domain-specific prior knowledge and reasoning capabilities, thereby addressing the challenges of unknown enzymes in real scientific discovery.

Microenvironment: pH 8.0, Tem 55°C

(1R,6R)-6-hydroxy-2-succinyl-cyclohexa-2,4-diene-1-carboxylate = 2-succinylbenzoate + H_2O

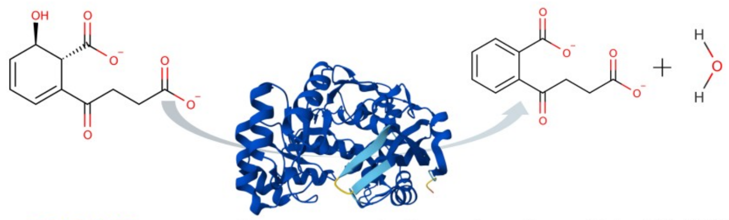

Function: EC:4.2.1.113 Enzyme: o-succinylbenzoate synthase Uniprot ID: B1A612

MPA-EC	EC:4.2.1.113.The enzyme exhibits maximum catalytic efficiency at pH 8.00 and 60.0°C. These optimal conditions are critical for the enzyme's structural stability and catalytic activity. The enzyme catalyzes: (1R,6R)-6-hydroxy-2-succinyl-cyclohexa-2,4-diene-1-carboxylate = 2-succinylbenzoate + H2O.
MPA-EC-Cold	EC:4.2.1.55. The enzyme exhibits maximum catalytic efficiency at pH 7.50 and 98.0°C. These optimal conditions are critical for the enzyme's structural stability and catalytic activity. The enzyme catalyzes: (R)-2-methyl-3-hydroxybutanoyl-CoA = (R)-2-methylbut-2-enoyl-CoA + H2O.
MPA-EC-Zero	EC:2.7.2.3;2.7.2.5. Explanation: The enzyme is a DNA polymerase. The DNA polymerase is a member of the DNA polymerase family, which is a large family of enzymes that are involved in the synthesis of new DNA strands. The enzyme has a high degree of specificity for its substrates, and its activity is optimal at a pH of around 7.0-8.0 and a temperature of around 37°C. The enzyme is also sensitive to inhibitors such as 2,6-dichloroindophenol and 2,6-dichloroindophenol. Based on the similarity sequence, catalytic reaction and the biochemical environment, the predicted EC number is EC:2.7.2.3;2.7.2.5.

Microenvironment: pH 7.4, Tem 37°C

(R)-2-hydroxyglutarate + A = 2-oxoglutarate + AH_2

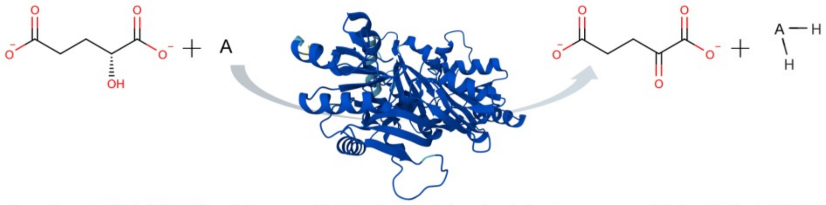

Function: EC:1.1.99.39 Enzyme: D-2-hydroxyglutarate dehydrogenase Uniprot ID: A4VGK4

MPA-EC	EC:1.1.99.39. The enzyme exhibits maximum catalytic efficiency at pH 7.50 and 45.0°C. These optimal conditions are critical for the enzyme's structural stability and catalytic activity. The enzyme catalyzes: (R)-2-hydroxyglutarate + A = 2-oxoglutarate + AH2.
MPA-EC-Cold	EC:1.1.99.40;1.1.2.4. The enzyme exhibits maximum catalytic efficiency at pH 8.50 and 85.0°C for (R)-lactate and 2 Fe(III)-[cytochrome c], catalyzing the reaction: (R)-lactate + 2 Fe(III)-[cytochrome c] = 2 Fe(II)-[cytochrome c] + pyruvate + 2 H(+). These substrates are critical for the enzyme's catalytic activity.
MPA-EC-Zero	EC:2.7.1.2;2.7.1.23. Explanation: According to the given sequence, it is a kinase enzyme. The kinase enzyme is a type of transferase enzyme that catalyzes the transfer of a phosphate group from a donor molecule to an acceptor molecule. The EC number for the kinase enzyme is EC 2.7.1.X, where X represents the specific type of kinase enzyme. Based on the biochemical environment and catalytic reaction, the sequence is most similar to Pyruvate kinase (EC 2.7.1.40) and Adenylate kinase (EC 2.7.1.51), but not exactly the same as the two enzymes. Therefore, the EC number is predicted to be EC 2.7.1.2;2.7.1.23.

Supplementary Fig. 6: Case study of model reasoning in cold-start and zero-shot conditions.

Supplementary Table 1. Sample data for fine-tuning.

<table>
<tr><th>Model</th><th>Fine-tuning data sample</th></tr>
<tr><td>MPA-EC-Initi</td><td>instruction:
Predict the EC number which consists of four digits from the following sequence, and the output format should be EC:X.X.X.X. A few sequences have two to four EC numbers. If you are certain that there are multiple labels, your output should be separated by ';' and the format should be EC:X.X.X.X;X.X.X.X
Input: Enzyme sequence:
MELPNIMHPVAKLSTALA...THTARMADKLR.
Output:
response:
EC:3.5.2.6.</td></tr>
<tr><td>MPA-EC-Rea</td><td>instruction:
Predict the EC number which consists of four digits from the following sequence, and the output format should be EC:X.X.X.X. A few sequences have two to four EC numbers. If you are certain that there are multiple labels, your output should be separated by ';' and the format should be EC:X.X.X.X;X.X.X.X
Input: Enzyme sequence:
MSTEHVDHKTIARFAEDK...KVFGSSFQG.
Considering the biochemical environment, such as catalytic reaction, let's think step by step.
Output:
response:
EC:2.7.7.85. The enzyme can catalyze the reactions: 3 ATP = 3',3',3'-c-tri-AMP + 3 diphosphate|2 ATP = 3',3'-c-di-AMP + 2 diphosphate, so the EC number of the enzyme is 2.7.7.85.</td></tr>
<tr><td>MPA-EC</td><td>instruction:
Predict the EC number which consists of four digits from the following sequence, and the output format should be EC:X.X.X.X. A few sequences have two to four EC numbers. If you are certain that there are multiple labels, your output should be separated by ';' and the format should be EC:X.X.X.X;X.X.X.X
The maximum similarity sequence is:
MRPSQRQA...LQKAALAGA, and the EC number of the maximum similarity sequence is: EC: 2.7.7.56. This may help in predicting the correct EC classification.
Input: Enzyme sequence:
MLIRAFIPAHIT...IYTERPKVGRWVG.
Considering the similarity sequence, biochemical environment, especially pH and temperature conditions, let's think step by step.
Output:
response:
EC:2.7.1.169. The enzyme exhibits maximum catalytic efficiency at pH 7.75 and 75.0°C. The enzyme catalyzes: (R)-pantoate + ATP = (R)-4-phosphopantoate + ADP + H(+). The maximally similar sequences tend to catalyze the same type of reaction and therefore may have the same EC numbers. The maximum similarity sequence has EC:2.7.7.56. Combining the information of the microenvironment, maximally similar sequence and the catalytic reaction, the EC number of the enzyme is 2.7.1.169.</td></tr>
</table>

Supplementary Table 2. Hyperparameter space of fine-tuning process.

Model	Hyperparameter name	Search space	Description
LoRA	r	8	LoRA rank size (low-rank matrix dimension)
	lora_alpha	32	LoRA scaling parameter (typically multiple of r)
	lora_dropout	0.1	LoRA dropout rate to prevent overfitting
Training	per_device_train_batch_size	8	Batch size per GPU
	num_train_epochs	2	Number of training epochs
	learning_rate	2e-5	Learning rate
	gradient_accumulation_steps	1	Gradient accumulation steps (to simulate larger batch size)
	weight_decay	0.01	Weight decay coefficient (L2 regularization)
	warmup_steps	100	Learning rate warm-up steps
	save_steps	500	Model saving interval steps
Optimizer	optim	adamw	Optimizer type (PyTorch AdamW)
	adam_beta1	0.9	AdamW optimizer β1 parameter
	adam_beta2	0.95	AdamW optimizer β2 parameter

Supplementary Table 3. Hyperparameter space of enzyme microenvironment prediction model.

Model	Hyperparameter name	Search space	Description
Training	batch_size	128	Batch size for training
	epochs	200	Number of epochs for Phase 1 and Phase 2
	learning_rate_nn	{5e-5 , 5e-6}	Learning rate for neural network
	weight_decay	1e-4	Weight decay coefficient
	gradient_clip	1.0	Gradient clipping threshold
Optimizer	optimizer	AdamW	Optimizer type
	scheduler	ReduceLROnPlateau	Learning rate scheduler
	scheduler_factor	0.7 (phase1), 0.8 (phase2)	Factor for learning rate reduction
	scheduler_patience	10 (phase1), 15 (phase2)	Patience for learning rate reduction
NN Module	input_dim	4096	Dimension of input features
	hidden_dim	{2048, 1024}	Hidden layer dimension
	dropout	0.1	Dropout probability
	dilations	{1, 2, 4, 8}	Dilation rates for dilated convolutions
ML Module Support Vector Regression (SVR)	kernel	'rbf' (all tasks)	Kernel type for Support Vector Regression
	C	pH: 20, tem: 200, OGT/TM: 100	Inverse of regularization strength
	epsilon	pH: 0.05, tem: 0.8, OGT/TM: 0.5	Epsilon-tube parameter
ML Module Random Forest (RF)	n_estimators	pH: 300, tem: 400, OGT/TM: 200	Number of trees in the forest
	max_depth	pH: 20, tem: 25, OGT/TM: 15	Maximum depth of the trees
	min_samples_split	pH: 2, tem: 2, OGT/TM: default	Minimum samples required to split a node
	tem_rf_depth	25	Maximum depth for temperature Random Forest
ML Module Gradient Boosting Regressor (GBR)	n_estimators	pH: 200, tem: 300, OGT/TM: 150	Number of boosting stages
	max_depth	pH: 10, tem: 12, OGT/TM: 8	Maximum depth of individual regression trees
	learning_rate	pH: 0.08, tem: 0.03, OGT/TM: 0.08	Learning rate for shrinkage

DNN Baseline for Disentangling Microenvironment Contributions

To isolate the contribution of microenvironment information from the LLM-based generative framework, we implemented a discriminative DNN baseline with a compact multi-scale CNN architecture, formulated as a multi-label classification framework for EC prediction. Microenvironment information was incorporated solely as an auxiliary multi-task learning objective during training. Importantly, during evaluation on NEW-392 and Price-149, no external microenvironment features were supplied to the DNN; the model relied exclusively on sequence-derived representations. This setting mirrors the inference procedure of MPA-EC, where microenvironment parameters are generated from the sequence itself, allowing us to isolate and measure the actual improvement brought by microenvironmental priors.

Two variants were trained: DNN w/o env (sequence features only) and DNN with env (sequence features + microenvironment auxiliary task), both using the exact same training set as MPA-EC. The results are shown in **Supplementary Tables 4** and **5**.

Supplementary Table 4. Performance comparison of DNN baseline models (with vs. without microenvironment auxiliary task) on the Price-149 dataset.

	DNN w/o env			DNN with env		
	P	R	F1	P	R	F1
Level 1	1.0000	1.0000	1.0000	1.0000	1.0000	1.0000
Level 2	0.5435	0.8929	0.6757	0.5682	0.8929	0.6944
Level 3	0.3524	0.6727	0.4625	0.3727	0.7455	0.4970
Level 4	0.0833	0.1623	0.1101	0.0912	0.1753	0.1200

Supplementary Table 5. Performance comparison of DNN baseline models (with vs. without microenvironment auxiliary task) on the NEW-392 dataset.

	DNN w/o env			DNN with env		
	P	R	F1	P	R	F1
Level 1	1.0000	1.0000	1.0000	1.0000	1.0000	1.0000
Level 2	0.6389	0.7857	0.6984	0.6286	0.8214	0.7146
Level 3	0.4419	0.6909	0.5390	0.5000	0.7455	0.5985
Level 4	0.1259	0.2208	0.1604	0.1271	0.2468	0.1678

Incorporating the microenvironment auxiliary task leads to an improvement in recall across both datasets. For example, on NEW-392, Level 3 recall increases from 0.6909 to 0.7455, indicating that the model becomes more capable of identifying true positive cases. This suggests that microenvironment information serves as a prior constraint, providing the model with more discriminative clues to accurately identify correct functional categories from a broader range of possibilities, thereby effectively reducing false negatives and expanding the model's coverage of the relevant functional space.

The overall performance of the DNN baseline (e.g., Level 4 F1 of 0.1200–0.1678) is comparable to that of early deep learning methods such as DeepEC, which is expected given its relatively simple

CNN architecture. In contrast, state-of-the-art methods such as CLEAN and GraphEC employ more sophisticated strategies including protein language model representations (e.g., ESM), contrastive learning, and structural information. These results indicate that the performance advantage of MPA-EC over the DNN baseline stems primarily from the generative reasoning capability of the LLM, rather than from the microenvironment features alone. Nevertheless, the consistent improvement from MPA-EC-Rea to MPA-EC-w/oMEMP to full MPA-EC in the main text confirms that microenvironment information, even when partially imputed, provides useful complementary constraints. The modest but consistent improvement from the auxiliary microenvironment task further confirms that microenvironmental information provides useful prior constraints that complement the sequence-based representations.

Supplementary Table 6. Performance comparison of DNN baseline models (sequence-only, full prompt, and without natural language description) on the Price-149 dataset.

	DNN only sequence			DNN full prompt			DNN w/o description		
	P	R	F1	P	R	F1	P	R	F1
Level 1	1.0000	1.0000	1.0000	1.0000	1.0000	1.0000	1.0000	1.0000	1.0000
Level 2	0.5435	0.8929	0.6757	0.5588	1.0000	0.7170	0.6944	0.8929	0.7812
Level 3	0.3524	0.6727	0.4625	0.2373	0.5185	0.3256	0.3824	0.7091	0.4968
Level 4	0.0833	0.1623	0.1101	0.0677	0.1667	0.0963	0.0868	0.1623	0.1131

Supplementary Table 7. Performance comparison of DNN baseline models (sequence-only, full prompt, and without natural language description) on the NEW-392 dataset.

	DNN only sequence			DNN full prompt			DNN w/o description		
	P	R	F1	P	R	F1	P	R	F1
Level 1	1.0000	1.0000	1.0000	1.0000	1.0000	1.0000	1.0000	1.0000	1.0000
Level 2	0.6389	0.7857	0.6984	0.6250	0.8929	0.7353	0.6190	0.9286	0.7429
Level 3	0.4419	0.6909	0.5390	0.4353	0.6727	0.5286	0.4588	0.7091	0.5571
Level 4	0.1259	0.2208	0.1604	0.1100	0.2078	0.1438	0.1327	0.2532	0.1741

To further isolate the contribution of generative reasoning from the input information itself, we designed an additional DNN baseline that receives exactly the same core information as MPA-EC, including sequence embeddings, Top-1 similar sequence embeddings with their EC labels, and reaction features, with the sole difference being discriminative classification versus generative reasoning. Three variants were compared: (1) DNN only sequence, using sequence features alone; (2) DNN full prompt, using the exact same natural language prompt as MPA-EC; and (3) DNN w/o description, removing textual noise and feeding only the core variables (sequence embeddings, Top-1 similar sequence embeddings with EC labels, and reaction features) into the DNN.

The results in Supplementary Tables 6 and 7 show that the DNN without natural language description achieves Level-4 F1 scores of 0.1131 on Price-149 and 0.1741 on NEW-392, only marginally higher than the sequence-only DNN (0.1101 and 0.1604). Notably, the full-prompt DNN underperforms even the sequence-only DNN, suggesting that natural language descriptions intended to guide the large language model can become distractions when presented repeatedly across examples. In contrast,

MPA-EC's generative framework effectively integrates these descriptions as part of the reasoning chain, achieving substantially higher performance (0.7037 and 0.7112). These results confirm that the performance advantage of MPA-EC over the DNN baselines is not merely due to access to additional information, but largely stems from its generative reasoning capability.

Homology-Reduced Evaluation

To assess the impact of homology leakage, we cleaned the training set using sequence similarity thresholds of 30% and 50% and retrained MPA-EC under these conditions. As shown in **Supplementary Table 8** and **9**, performance fluctuates as the threshold decreases, which we attribute to the high functional conservatism of enzyme sequences. Enzymes sharing the same EC number tend to maintain high sequence similarity due to evolutionary constraints. Applying a strict cutoff may remove most or even all samples of certain EC classes, inevitably compromising generalization.

We emphasize that MPA-EC does not simply copy EC numbers from retrieved similar sequences. For instance, in the prediction of Q11127, the retrieved sequences had ECs 2.4.1.152; 2.4.1.-; 2.4.1.65, while MPA-EC correctly predicted 2.4.1.152. In another case (A0A0A7AAW9 EC: 4.3.1.24), the retrieved sequence had EC 4.1.2.18, while MPA-EC correctly predicted 4.3.1.24, demonstrating reasoning rather than label copying.

All competing baselines were trained on the same dataset as MPA-EC, ensuring fair comparison. These results confirm that MPA-EC's performance relies on its generative reasoning capability rather than homology-based label copying, while also highlighting the inherent challenge of homology leakage in enzyme function prediction.

Supplementary Table 8. Performance comparison of MPA-EC under different sequence similarity thresholds on the Price-149 dataset.

	Full			Similar 50%			Similar 30%		
	P	R	F1	P	R	F1	P	R	F1
Level 1	1.0000	1.0000	1.0000	1.0000	1.0000	1.0000	1.0000	1.0000	1.0000
Level 2	1.0000	0.9474	0.9730	0.9000	0.9475	0.9231	0.9412	0.8421	0.8889
Level 3	0.9583	0.8519	0.9020	0.8519	0.8519	0.8519	0.8636	0.7037	0.7775
Level 4	0.7308	0.6786	0.7037	0.6230	0.6786	0.6496	0.5088	0.5179	0.5133

Supplementary Table 9. Performance comparison of MPA-EC under different sequence similarity thresholds on the NEW-392 dataset.

	Full			Similar 50%			Similar 30%		
	P	R	F1	P	R	F1	P	R	F1
Level 1	1.0000	1.0000	1.0000	1.0000	1.0000	1.0000	1.0000	1.0000	1.0000
Level 2	0.9021	0.9346	0.9204	0.9231	0.8519	0.8946	0.9057	0.8900	0.8929
Level 3	0.8966	0.9286	0.9123	0.9200	0.8421	0.8807	0.8889	0.8421	0.8649
Level 4	0.7597	0.6686	0.7112	0.7143	0.5429	0.6169	0.5786	0.4629	0.5143

Analysis of Retrieval Dependency and Potential Leakage

UniProt (2025_01) and BRENDA (Release 2024.1, December 2024) were downloaded in March 2025. To assess potential reaction-level leakage, we calculated the proportion of test samples where the Top-1 similar sequence shares the exact four-level EC match with the query. Since the retrieved reaction information is obtained through the EC numbers of similar sequences, and the EC system is a direct classification of enzyme-catalyzed reactions, the overlap in EC labels serves as a reasonable proxy for potential reaction-level overlap. On ECall (n=541), this proportion is 180/541 = 33.27%, indicating that for most test samples the nearest neighbor's EC label does not match the ground truth.

We further evaluated a "retrieval only" baseline that directly takes the EC number of the most similar sequence retrieved by MMseqs2 as the prediction, without model-based reasoning. As shown in **Supplementary Table 10**, this baseline achieves Level-4 F1 scores of 0.5472 (NEW-392) and 0.4464 (Price-149), substantially below MPA-EC's 0.7112 and 0.7037. A typical misclassification case is A0A075HNX4 (true EC 1.1.3.20): its top hit Q6CG88 (38.6% identity) has EC 1.1.3.37, while true same-EC homologs (Q9LW56, B5WWZ8) share only 28.8% and 29.2% identity and rank behind. Such cases are not uncommon, indicating that retrieval can be misled by functionally distinct but sequence-similar enzymes. Together, these analyses confirm that MPA-EC's predictions stem from generative reasoning rather than label copying or retrieval replication.

Supplementary Table 10. Comparison between the Retrieval Only baseline and MPA-EC across EC levels.

	NEW-392 dataset			Price-149 dataset		
	P	R	F1	P	R	F1
Level 1	1.0000	1.0000	1.0000	1.0000	1.0000	1.0000
Level 2	0.9630	0.9286	0.9455	1.0000	0.8421	0.9143
Level 3	0.9286	0.9123	0.9204	0.9091	0.7407	0.8163
Level 4	0.6364	0.4800	0.5472	0.4464	0.4464	0.4464

Supplementary Table 11. Progressive matching score and confidence score analysis for enzyme EC number annotation. "DS" group denotes the performance of sample groups categorized by EC number major or minor classes. For example, "DS4.1" represents all samples belonging to EC 4.1.X.X (carbon-carbon cleavage enzymes).

Group	Sample size	Progressive matching score	Confidence score
DS1	144	0.891	0.871
DS2	141	0.938	0.926
DS3	107	0.667	0.710
DS4	114	0.861	0.848
DS5	17	0.945	0.686
DS6	14	0.938	0.955
DS7	4	0.923	0.674
DS1.14	27	0.911	0.910
DS4.1	7	0.793	0.581

Supplementary Algorithm 1. Progressive matching score calculation for evaluating enzyme functional annotation performance.

Algorithm 1 Progressive Matching Score Calculation

Require: Actual EC number $A = a_1.a_2.a_3.a_4$, Generated EC number $G = g_1.g_2.g_3.g_4$, Decay factor $\lambda = 0.7$

Ensure: Normalized progressive matching score $S_{\text{norm}} \in [0, 1]$

// Initialize weights for each hierarchical level

$w_1 \leftarrow 1.0$ ▷ The mainclass level weight

$w_2 \leftarrow 0.7$ ▷ The subclass level weight

$w_3 \leftarrow 0.49$ ▷ The subsubclass level weight

$w_4 \leftarrow 0.343$ ▷ The designation level weight

$S \leftarrow 0$ ▷ Initialize total score

$S_{\max} \leftarrow 2.533$ ▷ Maximum possible score

// Calculate match scores for each level

for $i \leftarrow 1$ to 4 **do**

    if $a_i = g_i$ **then**

        $S \leftarrow S + w_i$

    end if

end for

// Normalize to [0,1] range

$S_{\text{norm}} \leftarrow S / S_{\max}$

return S_{norm}

Supplementary Algorithm 2. Confidence score calculation for evaluating enzyme functional annotation performance.

Algorithm 2 Confidence Score Calculation

Require: Set of n independent generations: $G = \{G_1, G_2, \ldots, G_n\}$, Each generation: $G_j = g_{j1}.g_{j2}.g_{j3}.g_{j4}$

Ensure: Confidence score $C \in [0, 1]$

```
// Check for insufficient generations
if n < 2 then
    return 0.0
end if
// Step 1: Calculate complete consistency (C_consistency)
freq ← empty dictionary                    ▷ To count generation frequencies
for j ← 1 to n do
    freq[G_j] ← freq[G_j] + 1
end for
max_freq ← max(freq.values)
C_consistency ← max_freq/n
// Step 2: Calculate average pairwise similarity (C_similarity)
total_similarity ← 0
pair_count ← 0
for j ← 1 to n − 1 do
    for k ← j + 1 to n do
        match_count ← 0
        for l ← 1 to 4 do
            if g_jl = g_kl then
                match_count ← match_count + 1
            end if
        end for
        similarity ← match_count/4
        total_similarity ← total_similarity + similarity
        pair_count ← pair_count + 1
    end for
end for
C_similarity ← total_similarity/pair_count
// Step 3: Weighted combination
C ← 0.4 × C_consistency + 0.6 × C_similarity
// Apply range constraints
if C < 0 then
    C ← 0
else if C > 1 then
    C ← 1
end if
return C
```
